# Comparison of dental findings between dentists and pediatricians using intraoral scan-based teledentistry in children

**DOI:** 10.1038/s41598-025-17870-w

**Published:** 2025-09-02

**Authors:** Nelly Schulz-Weidner, Eva May Schraml, Tina Frodermann, Norbert Krämer, Bernd Wöstmann, Maximiliane Amelie Schlenz

**Affiliations:** 1 Department of Pediatric Dentistry, Justus Liebig University Giessen, University Hospital Giessen, Schlangenzahl 14, 35392 Giessen, Germany; 2 Department of Oral and Maxillofacial Surgery, Justus Liebig University Giessen, University Hospital Giessen, Klinikstrasse 33, 35392 Giessen, Germany; 3 Department of Neuropediatrics, Justus Liebig University Giessen, University Hospital Giessen, Klinikstrasse 33, 35392 Giessen, Germany; 4 Department of Prosthodontics, Justus Liebig University Giessen, University Hospital Giessen, Schlangenzahl 14, 35392 Giessen, Germany; 5 Department of Prosthodontics, Christian Albrecht University of Kiel, University Hospital Schleswig-Holstein, Campus Kiel, Arnold-Heller-Strasse 3, 24105 Kiel, Germany

**Keywords:** Teledentistry, Intraoral scanners, Pediatric dentistry, Diagnosis, Remote consultation, Health care, Medical research

## Abstract

**Background:**

Asynchronous transmission of health information via teledentistry offers the potential for remote diagnosis in pediatric dentistry. The aim of this study was to compare teledental findings obtained from intraoral scans (IOS) with those from conventional visual examinations (VIS) in children. Specifically, the study assessed the diagnostic accuracy of teledentistry using IOS in evaluating oral health and determining treatment needs focusing on comparisons between dentists and pediatricians.

**Methods:**

Children (mean age 10.04 ± 2.90 years) underwent VIS during routine dental examinations. Two examiners performed the VIS, followed by digital IOS imaging of the oral cavity. Independent teledental evaluations based on the IOS data were then performed by a dentist (DEN) and a pediatrician (PED). Evaluation criteria included general dental status, presence of caries and molar incisor hypomineralization (MIH) (yes/no), restorations (yes/no; and type, if applicable), urgency of dental intervention, and treatment recommendations (no treatment, prophylaxis, follow-up, or immediate intervention). Agreement was analyzed using Gwet’s AC1, Cohen’s d, sensitivity, specificity, and area under the curve (AUC).

**Results:**

Almost perfect agreement (AC1 ≥ 0.81) was found for all test criteria, with two exceptions showing substantial agreement (AC1 = 0.61–0.80). Agreement values of overall dental status were 0.953/0.962 (primary dentition/permanent dentition (pD/PD)) for DEN and 0.908/0.923 for PED. Caries detection (yes/no) showed an agreement of 0.965/0.995 for DEN vs. 0.930/0.979 for PED, while restorations agreement was 0.988/0.993 (DEN) vs. 0.950/0.946 (PED). MIH assessment showed agreement of 0.996 (DEN) vs. 0.987 (PED). Cohen’s d for the comparison between DEN and PED ranged from small for MIH (0.17), caries detection (0.23) and overall dental status (0.34/0.35) to large for restoration type (0.89). The clinically most relevant item “urgency of dental intervention” showed almost perfect agreement (0.903 for DEN vs. 0.878 for PED), and the final treatment recommendations showed an almost perfect to substantial agreement of 0.832 (DEN) vs. 0.775 (PED). Notably, both examiners showed similar accuracy in assessing the urgency of intervention.

**Conclusions:**

This study demonstrates the potential of IOS-based teledentistry for pediatric dental assessments. The results indicate that pediatricians can effectively assess oral health and provide reliable treatment recommendations. This approach has the potential to increase access to dental care and to promote interdisciplinary collaboration in pediatric health care.

## Background

In contrast to general medicine, telemedicine is not really common in dentistry. This can be explained by several factors, such as a lack of rationale, political restrictions, or a lack of technology for appropriate teledentistry consultation. Teledentistry can be classified into two forms^1,2^ as real-time consultation with synchronous communication between patient and dentist using an audiovisual telecommunication technology^3,4^ and store and forward method with asynchronous transmission of health information using pre-recorded videos or digital images for later evaluation^5^.

Its application in pediatric dentistry is gaining momentum. Specialized pediatric telemedicine provides an opportunity for assessment, consultation, and management of dental conditions, especially when in-person care is hindered by distance or limited availability of dental specialists^6,7^. In this context, teledentistry is valuable not only for providing care to children who do not have easy access to dental facilities, but also for fostering improved interdisciplinary collaboration, particularly between dentists and pediatricians. This can contribute to the efficient allocation of resources and reduce unnecessary referrals and transport^2,8^. Teledentistry could therefore become a promising tool in dentistry, allowing for remote diagnosis and management of oral health^8,9^.

Ashtiani et al.^10^ demonstrated that teledentistry could serve as an alternative to analog visual examinations for caries detection in children. The integration of digital technologies such as intraoral cameras, electronic dental records, and communication tools has increased the accessibility of dental care, enabled remote consultation, and improved the overall delivery of dental health care^10–12^. However, one of the major concerns with telemedicine is the potential for misdiagnosis, which can lead to inappropriate treatment^13^. This is particularly relevant in pediatric care, where accurate diagnosis and treatment recommendations are crucial^14^.

Perhaps the skepticism of many dentists can be explained by the fact that there are few useful methods in dentistry today, because images taken with a cell phone or an intraoral camera or endoscope can only show small sections of the oral situation. Intraoral scanners, known from restorative dentistry, could provide a remedy^15^. Optimizing the quality of diagnostic images is essential, and intraoral scans (IOS) has emerged as an effective tool.

This is a completely new diagnostic option for patients, as intraoral scanners have never before been used for such an approach. Intraoral scanners take single images and align them into a color 3D data set of the intraoral situation. They have been used in dentistry for many years for digital impression taking in the digital workflow for manufacturing dental restorations. IOS devices, which already generate digital three-dimensional dental models, are increasingly being used in clinical practice as diagnostic tools, providing quantitative data that can complement visual assessments. This technology has the potential to improve diagnostic accuracy to the benefit of both clinicians and patients^16^.

In addition, the range of indications is expanding, and the technological developments allow even laptop versions of intraoral scanners that able an easy transport and use. The application is not limited to dentists, as IOS can also be taken by trained non-dental professionals (e.g., pediatricians).

In the context of pediatric dentistry, a critical question is how different health care professionals, namely dentists and pediatricians, interpret and act on teledental data. Comparing the accuracy, reliability, and effectiveness of teledental diagnoses made by both groups is essential to understanding the potential of teledentistry in this interdisciplinary setting. Sensitivity (true positive/all positive findings) and specificity (true negative/all negative findings) are traditional measures used to evaluate diagnostic quality, and these will be critical in determining whether dentists and pediatricians can make equally reliable diagnostic and management decisions using teledentistry^17^. The following null hypotheses were tested: 1. There is no significant difference between digital teledental findings (IOS) and the gold standard of analog visual examination (VIS). 2. There is no significant difference in the diagnostic reliability between dentists and pediatricians. 3. Sensitivity and specificity of digital teledental assessments (IOS) do not significantly differ from those of analog visual examination (VIS), regardless of whether assessments are conducted by dentists or pediatricians.

## Methods

The study was conducted according to the guidelines of the Declaration of Helsinki, approved by the Ethics Committee of the Medical Faculty of the Justus Liebig University (JLU, ref. no. 46/20).

### Study group

Sample size calculation was done using the formula described by Bujang et al. based on the expected diagnostic performance for the IOS^18^. With a target power of 90% and an alpha value of 0.05, 70 participants were calculated as necessary.

All children aged 4–17 years who underwent regular dental examination at the Department of Pediatric Dentistry at JLU (Germany) between August 2022 and February 2023 participated in this study. Therefore, the participants comprised a representative cross-section of patients treated at the pediatric dentistry department of a university hospital. Common treatment measures included the management of dental caries and structural anomalies, preventive interventions, trauma care, treatment under general anesthesia, specialized care for children with special healthcare needs, and thorough diagnostic assessments.

In addition to a thorough verbal explanation of the study procedures, written informed consent was obtained from all parents/guardians.

### Standardization, training, calibration, and blinding

To ensure comparable test conditions and obtain reproducible data based on predefined criteria, each patient was initially examined by two experienced dentists (N.S.-W. and M.A.S.) using an analog method, with the visual examination (VIS) defined as the reference method and gold standard for subsequent analysis. The IOS required for teledental diagnosis were also performed by the same examiner. Subsequently, the teledental digital findings of the IOS were evaluated by two independent examiners: one dentist (DEN, E.S.) and one pediatrician (PED, T.F.).

Prior to the study, E.S. and T.F. were trained and calibrated by senior researchers (N.S.-W. and M.A.S.). The training period lasted two weeks and consisted of both theoretical and practical parts. The theoretical part included structured instruction on the clinical features and diagnostic criteria of the conditions under study, using standardized materials and case examples (e.g., following the approach described by Kühnisch et al.^19^). The practical part involved supervised clinical assessments of ten pediatric patients under the guidance of N.S.-W. and M.A.S. To ensure diagnostic consistency, readiness for independent assessment was evaluated based on each examiner’s performance in a benchmark assessment, including both intra-rater reliability (agreement within the same examiner) and inter-rater reliability (agreement with expert diagnoses)^19^. According to the classification by Landis and Koch^20^, the level of agreement was almost perfect, with a Cohen’s kappa coefficient (κ) of 0.95. Only after achieving this benchmark were E.S. and T.F. permitted to conduct independent assessments within the study. While E.S. received extended training aimed at reaching a diagnostic level comparable to that of the senior dental researchers (M.A.S. and N.S.-W.), T.F. as pediatrician, received tailored instruction focused on core dental diagnostic skills. This included basic knowledge of caries recognition, structural dental anomalies (e.g., MIH), differentiation between primary and permanent dentition, and identification of soft tissue signs such as swelling, redness, and plaque accumulation. It is important to acknowledge that, despite this structured theoretical and practical training, a pediatrician cannot attain the diagnostic proficiency of a trained dentist. Nonetheless, this tailored training allowed for a realistic evaluation of the potential role of pediatricians in supporting teledentistry-based screening or triage in pediatric patients.

### Visual examination (VIS)

The presence of caries was verified as yes/no decision (dentin caries) under standardized lighting (25,000 lx), with a mouth mirror and an air syringe. Both primary dentition (pD) and permanent dentition (PD) were included in the evaluation.

### Intraoral scans (IOS)

**Fig. 1:**
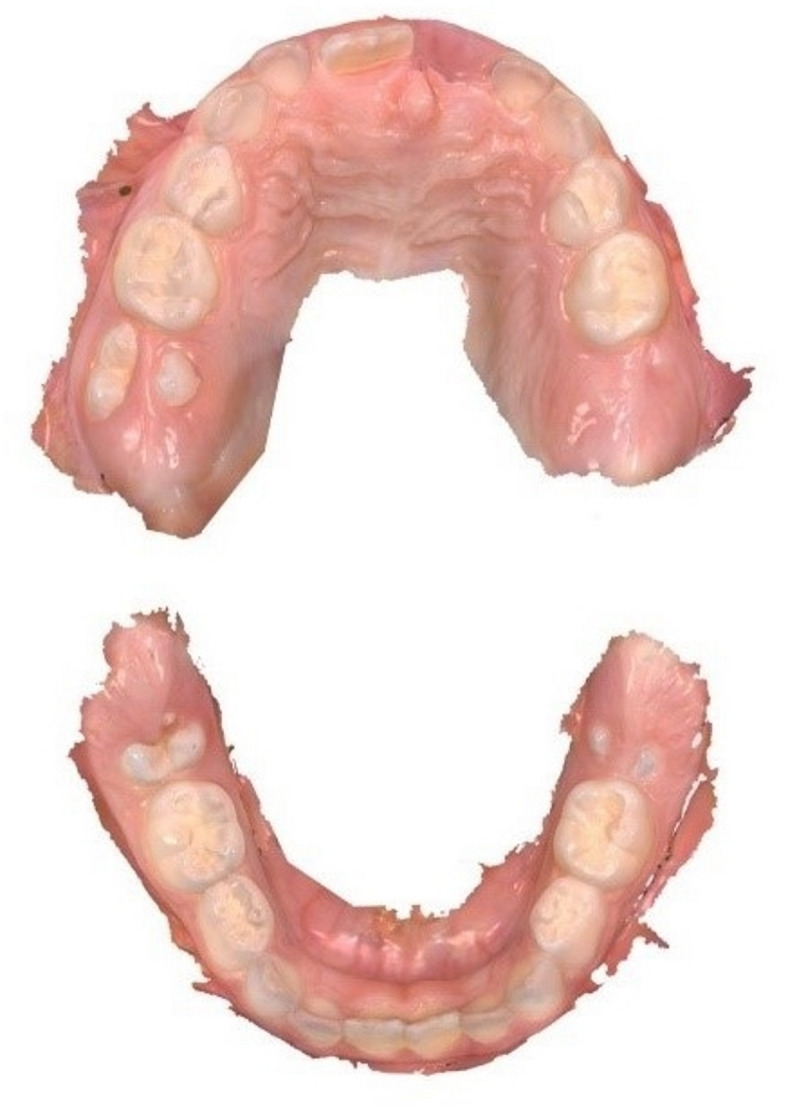
Example of intraoral scans (IOS) of the upper and lower jaw of a six years old child with erupting first permanent molars #16, #36 and #46 (FDI scheme) and upper front teeth #11. Fissure sealings in teeth #55, #65, #75 and #85 and glass ionomer cement restoration in teeth #54 and #64.

The IOS were captured by Trios 4 (software version 20.1.4, 3Shape, Copenhagen, Denmark) that was used to scan the entire upper and lower jaw following a standardized scanning path, which included the occlusal, oral, and buccal surfaces. Prior to scanning, the intraoral scanner was calibrated according to the manufacturer’s instructions. Figure 1 displays an example of IOS.

**Fig. 2:**
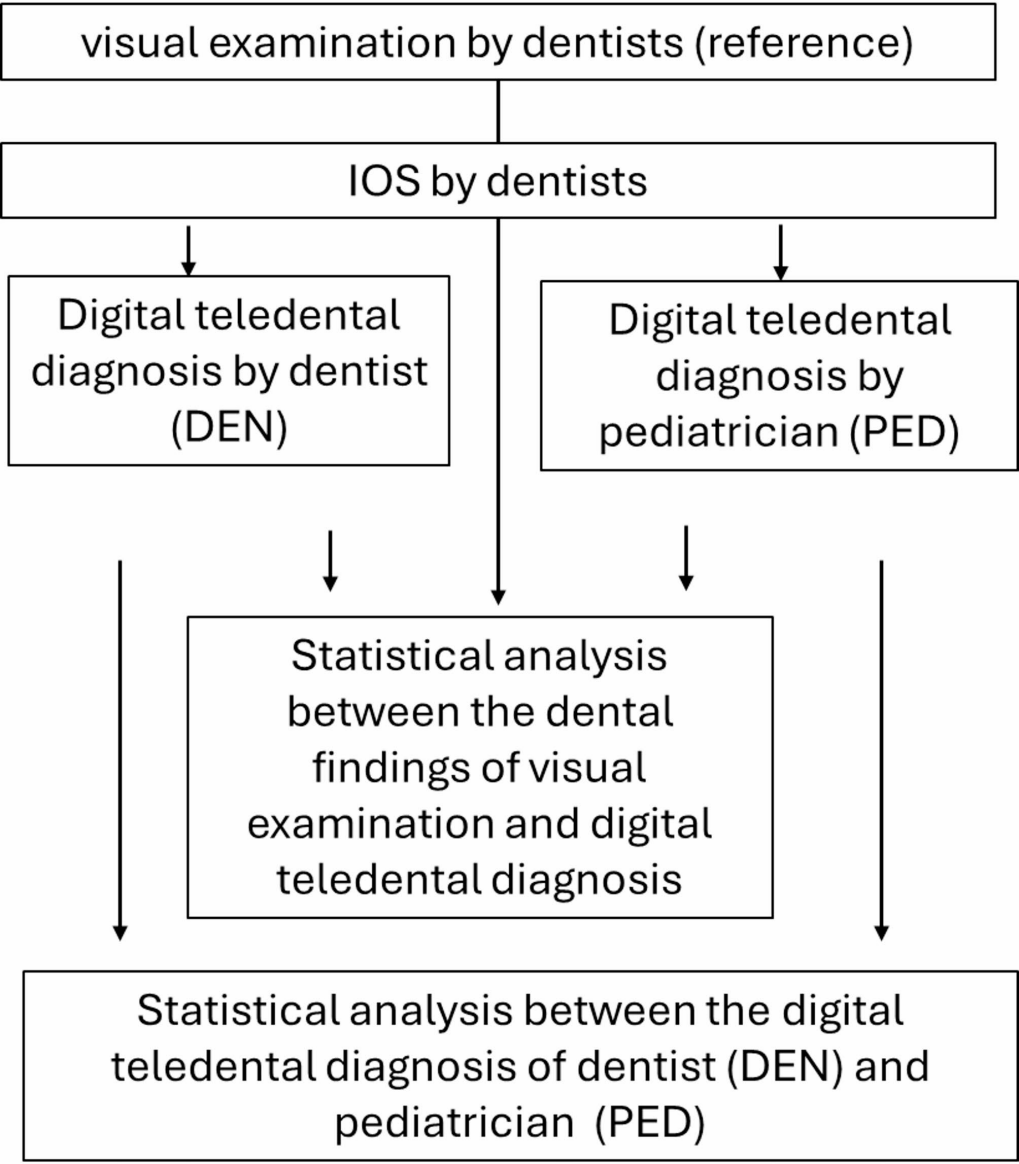
Flow scheme of the investigation (IOS = intraoral scans).

To ensure standardized comparability between the established analog VIS of the oral cavity, considered the gold standard and reference method, and the new digital teledental diagnosis based on IOS, a diagnostic sheet was developed to record the following aspects of oral health: overall dental status, including caries (yes/no), restorations (yes/no) and if yes, type of restorations (fillings materials (composite/glass ionomer cements), fissure sealing, steel crown), and molar incisor hypomineralization (MIH). Additionally, urgency of dental intervention (urgent/not urgent) and treatment recommendations (no treatment/prophylaxis/check-up for suspected caries lesions/immediate dental intervention required) were also recorded. Figure 2 shows the study procedure.

### Reliability

To compare the digital (IOS) and analog (VIS) findings, Gwet’s AC1 (κ) was used. Kappa values were categorized as poor agreement (< 0.00), slight agreement (0.00–0.20), fair agreement (0.21–0.40), moderate agreement (0.41–0.60), substantial agreement (0.61–0.80), and almost perfect agreement (≥ 0.81)^20^. Additionally, Cohen’s d, a measurement of effect size (d = 0.2: small, d = 0.5: medium, d = 0.8: large), was used to assess significant differences between the two digital examiners, DEN and PED^21^ in terms of caries detection (pD and PD), restorations in general and type of restorations (pD and PD), MIH, urgency of dental intervention and treatment recommendation.

### Sensitivity and specificity

To assess the diagnostic quality of the digital findings (IOS) in comparison to the analog findings (VIS), sensitivity (SE), specificity (SP), and area under the curve (AUC) values were calculated using receiver operating characteristic (ROC) analysis for caries detection and restorations (pD and PD), MIH and urgency of dental intervention. Higher AUC values indicate better diagnostic quality^22^.

### Statistical analysis

SPSS Statistics (version 28, IBM, Armonk, NY, USA) was used for the statistical analysis. Due to the study design of an examination on a tooth level, data was further analyzed separately for pD and PD.

## Results

A total of 70 patients (42 males and 28 females) with a mean age of 10.04 ± 2.90 years (range 4–17 years) were included in this study. A total of 1746 teeth were examined, consisting of 587 primary teeth (pD) and 1159 permanent teeth (PD).

### Reliability

For the overall dental status, the digital teledental evaluation with IOS showed almost perfect agreement with the reference method (VIS), in both dentitions (pD/PD) for DEN (κ = 0.953/0.962) and for PED (κ = 0.908/0.923).

Specifically, the presence of caries was detected almost perfectly for both dentitions (pd/PD), with DEN (κ = 0.965/0.995) and PED (κ = 0.930/0.979). Restorations were also recognized almost perfectly in both dentitions (pD/PD) for DEN (κ = 0.988/0.993) and PED (κ = 0.950/0.946). The type of restorations achieved perfect agreement values of κ = 1.000 for DEN vs. κ = 0.965 for PED for pD, similarly good values were obtained for the PD (DEN: κ = 0.960 vs. PED: κ = 0.941).

MIH was detected almost perfectly by both examiners (DEN/PED: κ = 0.996/0.987).

The urgency of dental intervention showed almost perfect agreement with κ = 0.903 for DEN and κ = 0.878 for PED. For the final treatment recommendation, almost perfect agreement was observed with κ = 0.832 between DEN and the gold standard, while there was significant agreement with κ = 0.775 between PED and the gold standard (Table 1).

**Table 1:** Reliability of detailed diagnostics for digital teledental diagnosis (IOS) in comparison to referent method visual examination (DEN: dentist, PED: pediatrician).

Items	DEN (ĸ)	PED (ĸ)
Caries in primary dentition	0.965	0.930
Caries in permanent dentition	0.995	0.979
Restorations in primary dentition	0.988	0.950
Restorations in permanent dentition	0.993	0.946
Type of restorations primary dentition	1.000	0.965
Type of restorations in permanent dentition	0.960	0.941
Molar incisor hypomineralization (MIH) (only for permanent dentition)	0.996	0.987
Urgency of dental intervention (pooled data of primary and permanent dentition)	0.903	0.878
Treatment recommendation (pooled data of primary and permanent dentition)	0.832	0.775

Cohen’s effect size analysis revealed weak effect strength between the observers DEN and PED for MIH (d = 0.17), caries detection (d = 0.23) and overall dental status (d = 0.34 primary, d = 0.35 permanent). However, a strong effect (d = 0.89) was noted for type of restorations, indicating significant inter-observer differences (DEN better than PED).

### Sensitivity and specificity

**Fig. 3:**
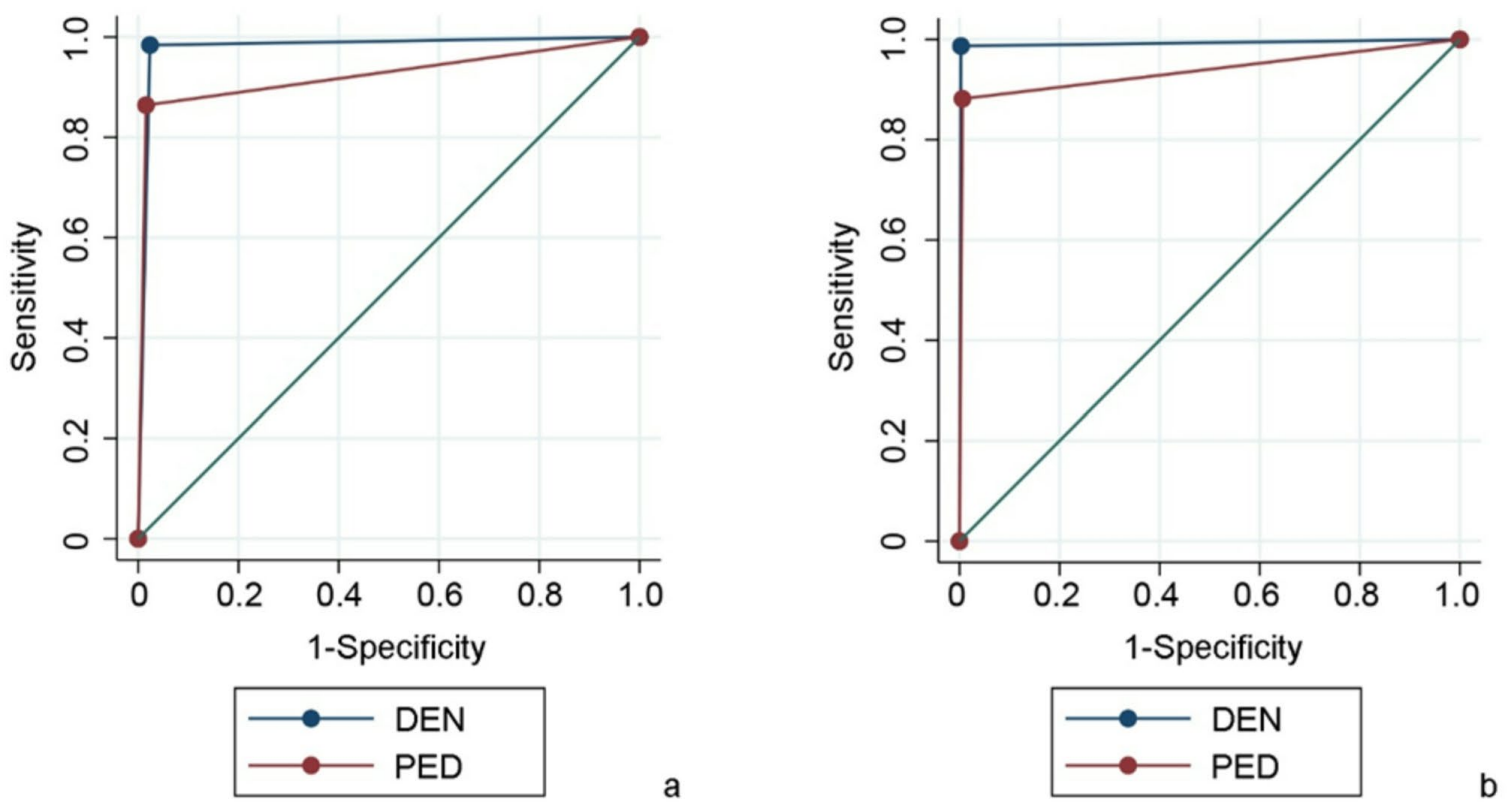
Receiver operating characteristic (ROC) curve analysis for caries detection in primary (**a**) and permanent (**b**) teeth for dentist (DEN) and pediatrician (PED).

For caries detection in primary teeth, DEN showed higher sensitivity (0.984/AUC 0.980 vs. 0.865/AUC 0.924) while PED demonstrated higher specificity (0.984 vs. 0.976). Similar trends were observed in permanent teeth: DEN (SE/SP/AUC: 0.987/0.997/0.992) vs. PED (0.883/0.994/0.938; Table 2, Fig. 3). Even though both observers reached high sensitivity and specificity, DEN detected significantly more caries lesions in both primary (*p* = 0.002) and permanent (*p* = 0.023) teeth compared to PED.

**Table 2:** Digital teledental diagnosis regarding sensitivity, specificity, and area under curve (AUC) values for dentist (DEN) and pediatrician (PED).

Items	DEN			PED			
	Sensitivity	Specificity	AUC	Sensitivity		Specificity	AUC
Caries in primary dentition	0.984	0.976	0.980	0.865		0.984	0.924
Caries in permanent dentition	0.987	0.997	0.992	0.883		0.994	0.938
Restorations in primary dentition	0.919	0.997	0.958	0.539		1.000	0.783
Restorations in permanent dentition	0.973	1.000	0.986	0.779		1.000	0.885
Molar incisor hypomineralization (MIH) (only for permanent dentition)	0.997	0.992	0.995	0.998		0.870	0.933
Urgency of dental intervention (pooled data of primary and permanent dentition)	0.980	0.857	0.918	0.959		0.857	0.908

**Fig. 4:**
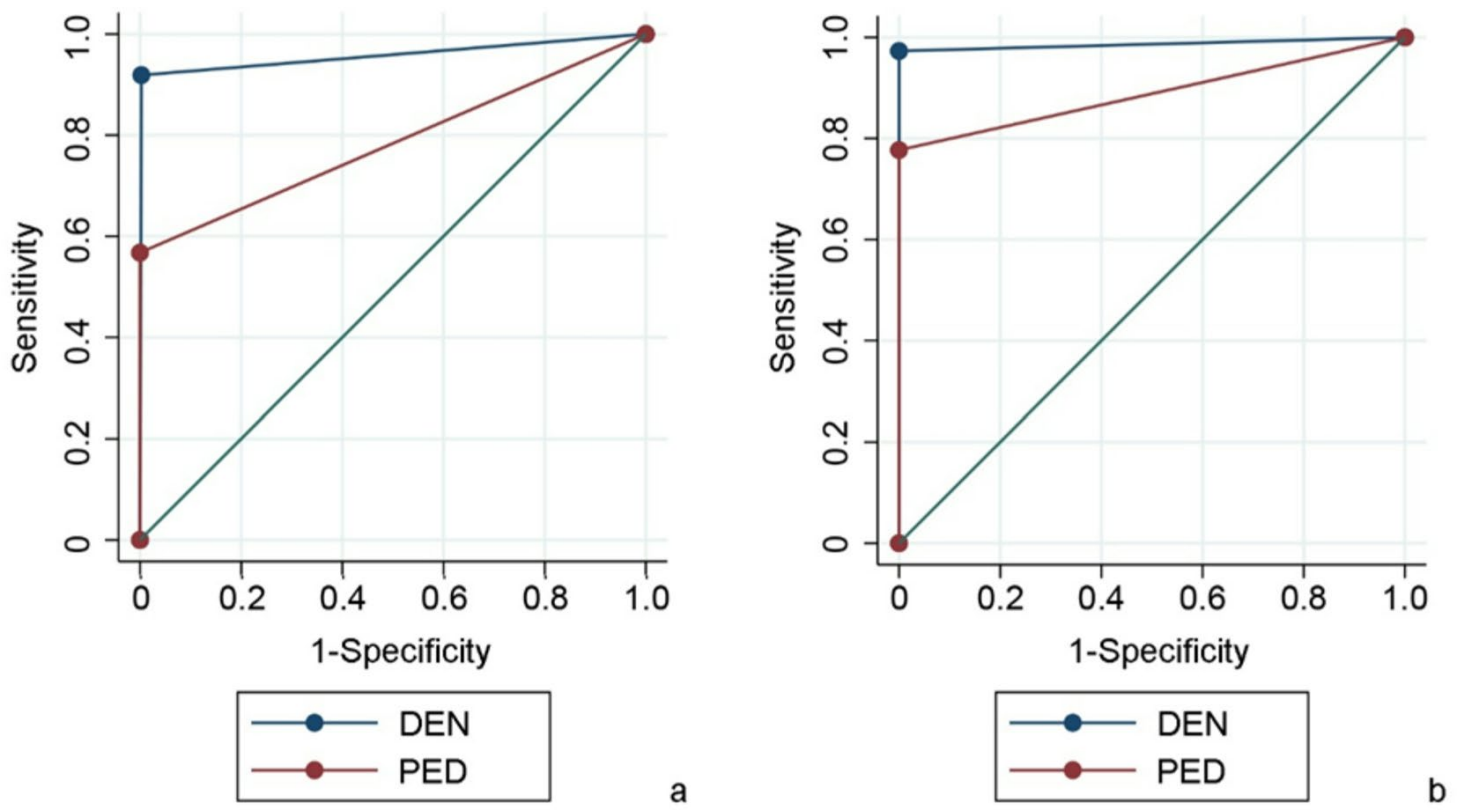
Receiver operating characteristic (ROC) curve analysis for restorations in primary (**a**) and permanent (**b**) teeth for dentist (DEN) and pediatrician (PED).

Regarding restorations, DEN exhibited highest sensitivity, specificity and AUC values for detection of restorations in both primary (0.919/0.997/0.958) and permanent teeth (0.973/1.000/0.986) followed by PED, demonstrating lower values for SE in pD (SE/AUC 0.539/0.783) and PD (0.779/0.885) and similar values for SP for both dentitions (1.000/1.000; Table 2, Fig. 4).

Even though both observers reached high specificity, DEN detected significantly more restorations than PED in primary and permanent teeth (*p* = 0.000), which is supported by higher SE values.

MIH was detected with comparable sensitivity in DEN (0.997) vs. PED (0.998), whereas specificity was higher detected in DEN (0.992; AUC 0.995 vs. PED 0.870; AUC 0.933; Table 2). DEN detected significantly more MIH, than PED (*p* = 0.000).

**Fig. 5:**
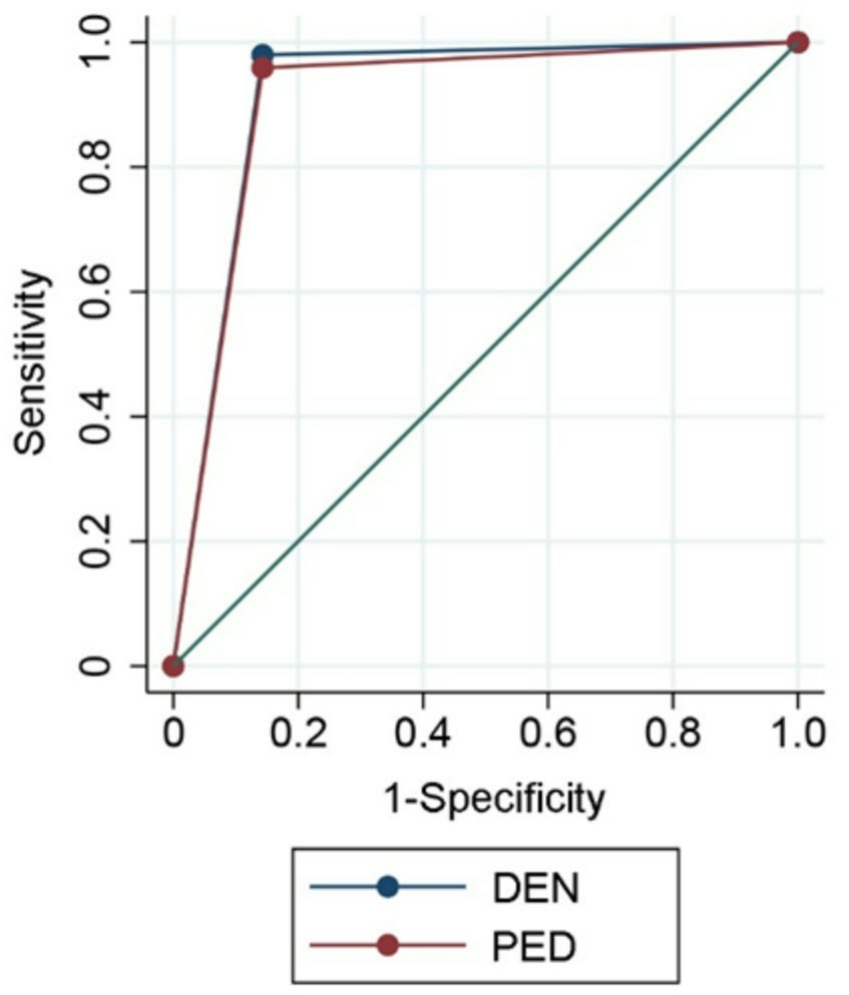
Receiver operating characteristic (ROC) curve analysis for urgency of dental intervention for dentist (DEN) and pediatrician (PED).

With regard to urgency of dental intervention, DEN showed comparable sensitivity, specificity and AUC (SE, SP, AUC; 0.980/0.857/0.918) to PED (0.959/0.857/0.908; Table 2, Fig. 5). There was no significant difference between the two digital examiners (*p* = 0.777).

## Discussion

Although the first null hypothesis that there is no significant difference between digital teledental findings and the gold standard of analog visual examination had to be rejected, the results of the present study demonstrate a high level of agreement between the reference method of visual examination (VIS) and the teledentistry approach based on intraoral scans (IOS), emphasizing the suitability of IOS for determining dental status. This high reliability is particularly noteworthy when comparing the results obtained by a dentist (DEN) and a pediatrician (PED), underscoring the feasibility of using IOS in both dental and non-dental professionals. However, while IOS are not yet routinely used in clinical settings for documentation purposes alone, their use in teledentistry provides an opportunity to improve the diagnostic skills of non-dental professionals. Ashtiani et al. were already able to demonstrate comparable results in the visual clinical examination of the oral cavity and the collection of dental findings with the help of dentists and community health workers. However, mobile phone photographs were used for this purpose^10^.

A key aspect of this study was the comparison between dentists (DEN) and pediatricians (PED) in the interpretation of teledentistry findings. While PEDs showed almost perfect agreement with VIS in assessing overall oral health parameters, such as urgency of dental intervention and corresponding treatment recommendations, certain more detailed diagnostic elements, such as identification of restoration types, were assessed less accurately (κ = 0.72). Consequently, the second null hypothesis – that there is no difference in the reliability of findings between DENs and PEDs – was rejected. Nevertheless, the results indicate that PEDs are capable of reliably assessing overall oral health status and making appropriate treatment decisions. The observed limitations in the assessment of more complex dental conditions did not significantly affect the accuracy of the final treatment recommendations derived from the IOS-based assessments.

The high level of agreement between DEN and PED in assessing the urgency of dental intervention (κ = 0.90 for DEN and κ = 0.88 for PED) suggests that, with appropriate training, pediatricians are well positioned to play an important role in teledentistry – particularly in scenarios that require timely dental evaluations, such as preoperative assessments or when oral health may affect other medical treatments. This approach has the potential to streamline care pathways for vulnerable pediatric populations, reduce wait times, and help prevent the progression of oral disease that might otherwise require more invasive interventions.

Training is a critical factor contributing to this high level of agreement. In this study, all examiners, including DEN and PED, underwent thorough calibration and pre-examination training, a practice also highlighted by Hung et al.^23^. This highlights that while DEN may be more accurate in assessing complex dental problems, PED can still reliably assess the most urgent aspects of oral health and contribute to care coordination when adequately trained. The findings suggest that teledentistry allows PEDs, who typically lack specific dental training, to expand their role in dental diagnosis and decision making, facilitating interdisciplinary care and better patient outcomes. A limitation of this study is that only a single pediatrician was trained and involved in the diagnostic assessment, which may limit the generalizability of the findings. In addition, the study was conducted at a single center, which may further limit the external validity of the results.

The findings also point to an important opportunity for non-dental professionals, such as PEDs, to become more involved in early oral health assessments. This could be particularly beneficial in cases where PEDs are already managing other health conditions, such as congenital heart defects or immunocompromised states, and need to ensure that dental health is addressed prior to other medical procedures. Studies such as that of Estai et al.^8^ have already demonstrated the importance of involving non-dental professionals in the identification of children at risk, particularly in underserved areas where access to dental specialists may be limited. However, it should also be noted that the purchase of an intraoral scanner is costly.

Although there were some differences in the accuracy of specific findings between DEN and PED, particularly for more detailed diagnostic aspects, which leads to the rejection of our third null hypothesis that there is no difference in terms of sensitivity and specificity between digital teledental findings (IOS) and the gold standard of analog visual examination (VIS), when assessed by dentists and pediatricians, it must be pointed out that the overall clinical impact was minimal. This supports the potential of IOS-based teledentistry to improve the overall quality of care and make dental assessments more accessible, especially when resources are limited. The ability of pediatricians to assess the urgency of dental conditions and make appropriate recommendations could improve the speed and coordination of care for children requiring dental intervention, particularly in the hospital setting.

While the ability of PEDs to identify complex dental conditions may be somewhat limited compared to a DEN, their involvement in early screening and triage could significantly improve patient care. As highlighted in previous studies, effective training and calibration programs for non-dental professionals are essential for the accurate performance of oral health assessments to ensure that data obtained from teledentistry can be reliably used to guide treatment decisions^24^.

In summary, while DEN remains the gold standard for the diagnosis and management of complex dental problems, appropriately trained PEDs can play a valuable role in the early detection of dental disease and the coordination of care. The integration of teledentistry into pediatric practice offers a promising avenue for improving access to dental diagnosis and care, particularly for vulnerable populations^25^. The potential for collaboration between DEN and PED through teledentistry could lead to better outcomes, more efficient treatment planning, and a more holistic approach to pediatric health care.

## Conclusions

This study highlights the significant potential of IOS-based teledentistry in pediatric dental care. The results show that both dentists and pediatricians can effectively assess children’s oral health using intraoral scans, allowing for accurate assessment of treatment urgency. This approach supports more efficient treatment planning, which can help reduce both procedural risks and the extent of necessary interventions. While the results indicate that both dentists and pediatricians can provide reliable assessments, differences were observed in the assessment of specific dental conditions, such as the identification of restoration types. Nevertheless, the method remains highly valuable in promoting interdisciplinary collaboration, particularly in settings with limited access to specialized dental care. Implementation of IOS-based teledentistry could improve coordination among health care providers, expand access to dental diagnosis, and contribute to improved oral health outcomes for vulnerable pediatric populations.

## Data Availability

The datasets in this article are available from the corresponding author upon reasonable request.

## Abbreviations

AUC: Area under curve
MIH: Molar incisor hypomineralisation
DEN: Dentist
PED: Pediatrician
ROC: Receiver operating characteristic
pD: Primary dentition
PD: Permanent dentition
VIS: Visual examination
IOS: Intraoral scans
SE: Sensitivity
SP: Specificity
